# Shedding Light on a Secretive Tertiary Urodelean Relict: Hynobiid Salamanders (*Paradactylodon persicus* s.l.) from Iran, Illuminated by Phylogeographic, Developmental, and Transcriptomic Data

**DOI:** 10.3390/genes10040306

**Published:** 2019-04-18

**Authors:** Matthias Stöck, Fatemeh Fakharzadeh, Heiner Kuhl, Beata Rozenblut-Kościsty, Sophie Leinweber, Riddhi Patel, Mehregan Ebrahimi, Sebastian Voitel, Josef Friedrich Schmidtler, Haji Gholi Kami, Maria Ogielska, Daniel W. Förster

**Affiliations:** 1 Leibniz-Institute of Freshwater Ecology and Inland Fisheries (IGB), Müggelseedamm 301, D-12587 Berlin, Germany; kuhl@igb-berlin.de (H.K.); solein@pivko.de (S.L.); 2 Department of Biology, Faculty of Sciences, Shahid Chamran University of Ahvaz, Ahvaz 61357-43135, Iran; ffakharzadeh2015@gmail.com; 3 Department of Evolutionary Biology and Conservation of Vertebrates, Wroclaw University, Sienkiewicza 21, 50-335 Wroclaw, Poland; beata.rozenblut-koscisty@uwr.edu.pl (B.R.-K.); maria.ogielska@uwr.edu.pl (M.O.); 4 Evolutionary Genetics Department, Leibniz-Institute for Zoo and Wildlife Research, Alfred-Kowalke-Str. 17, 10315 Berlin, Germany; riddhibio27@gmail.com (R.P.); foerster@izw-berlin.de (D.W.F.); 5 Department of Biology, College of Sciences, Shiraz University, Shiraz 71467-13565, Iran; m.ebrahimi@shirazu.ac.ir; 6 School of Biological Sciences, Flinders University, Adelaide, SA 5001, Australia; 7 Independent Researcher, Spangenbergstraße 81, D-06295 Eisleben, Germany; sebastian.voitel@t-online.de; 8 Zoologische Staatssammlung, Münchhausenstraße 21, 81247 München, Germany; josef@schmidtler.eu; 9 Department of Biology, Faculty of Sciences, Golestan University, Gorgan 49136-15759, Iran; hgkami2000@yahoo.com

**Keywords:** Urodela, Hynobiidae, phylogeography, RNAseq, genomics, gene expression, gonadal development, histology, systematics

## Abstract

The Hyrcanian Forests present a unique Tertiary relict ecosystem, covering the northern Elburz and Talysh Ranges (Iran, Azerbaijan), a poorly investigated, unique biodiversity hotspot with many cryptic species. Since the 1970s, two nominal species of Urodela, Hynobiidae, *Batrachuperus* (later: *Paradactylodon*) have been described: *Paradactylodon persicus* from northwestern and *P. gorganensis* from northeastern Iran. Although *P. gorganensis* has been involved in studies on phylogeny and development, there is little data on the phylogeography, systematics, and development of the genus throughout the Hyrcanian Forests; genome-wide resources have been entirely missing. Given the huge genome size of hynobiids, making whole genome sequencing hardly affordable, we aimed to publish the first transcriptomic resources for *Paradactylodon* from an embryo and a larva (9.17 Gb RNA sequences; assembled to 78,918 unigenes). We also listed 32 genes involved in vertebrate sexual development and sex determination. Photographic documentation of the development from egg sacs across several embryonal and larval stages until metamorphosis enabled, for the first time, comparison of the ontogeny with that of other hynobiids and new histological and transcriptomic insights into early gonads and timing of their differentiation. Transcriptomes from central Elburz, next-generation sequencing (NGS) libraries of archival DNA of topotypic *P. persicus*, and GenBank-sequences of eastern *P. gorganensis* allowed phylogenetic analysis with three mitochondrial genomes, supplemented by PCR-amplified mtDNA-fragments from 17 museum specimens, documenting <2% uncorrected intraspecific genetic distance. Our data suggest that these rare salamanders belong to a single species *P. persicus* s.l. Humankind has a great responsibility to protect this species and the unique biodiversity of the Hyrcanian Forest ecosystems.

## 1. Introduction

The Caspian or Hyrcanian Forests present a unique Tertiary relict ecosystem that mostly covers the northern and few interior chains of the Elburz Range and the Talysh Mountains (Iran, Azerbaijan) as well as their western adjacent ranges [1,2]. Together with the Colchic broadleaf forests of Georgia, the Hyrcanian Forests that in part even cover the southern Caspian coastal plain, constitute the most important refugia and the last relics of primary temperate deciduous broad-leaved forests worldwide [3]. They have been almost poetically called “the crib” of the Central European woods [1], and while this is true regarding their function as a Pleistocene refugium for some temperate species, its prominence for global biodiversity and conservation may be better highlighted by their importance and uniqueness as a Tertiary relict ecosystem. Despite biogeographic connections to the Colchis, both regions became geographically isolated following the uplift and folding of the Lesser Caucasus during the Palaeocene–Miocene and subsequent volcanic uplift during the Pliocene–Quaternary [4,5]. Thus, it is not very surprising that the Hyrcanian Forests as an ancient ecosystem harbor many endemic species, with numerous plants [1,3,5] and animals [2], and also comprise a great cryptic biodiversity [6] that still remains to be discovered.

With respect to vertebrates, the Hyrcanian Forests are inhabited by some of the most western living representatives of the ancient urodelan family Hynobiidae (only *Salamandrella keyserlingii* reaches more western latitudes in northern Eurasia). These salamanders belong to the genus *Paradactylodon* Risch, 1984 [7] (previously *Batrachuperus*). To date, they comprise in Iran two nominal taxa, *Paradactylodon persicus* and *P. gorganensis* (details below). Their original descriptions were not comparable as they were based on the morphology of two different life stages (larvae vs. adults). Specifically, only merely 50 years ago, the Persian mountain salamander has been originally described as *Batrachuperus persicus* Eiselt & Steiner, 1970 [8]. Due to the secretive lifestyle of the species, its description was based on five larvae, collected near Assalem in the Talysh Mountains of the Gilan province of northwestern Iran. In 1971, J.J. and J.F. Schmidtler [9] collected some larvae from the type locality (“topotypic larvae”). After their metamorphosis in captivity, a brief description of the juvenile salamanders was presented [9].

**Figure 1:**
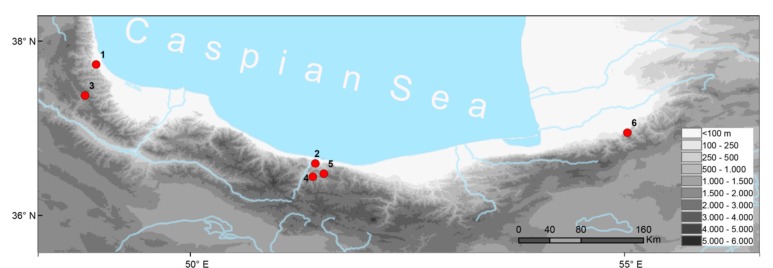
Map with sampling localities. **1**–Gilan Province, Talysh-mountains, 12 km S Assalem, 700 m a.s.l.; **2**–Mazandaran Prov., SE of Chalous city, Lashkenar village, valley of Zereshkdarreh; **3**–Yeilagh-e-Sarasi, ca. 45 km SE Khalkhal, Delmadeh (Daylamdeh) village; **4**–Mazandaran Province, near Veysar village; **5**–Iran, Mazandaran Province, Veysar village, Noshahr City, Zaresk-Dareh; **6**–Shirabad Cave, 5 km SE (by air) of Shirabad, 60 km E (by air) of Gorgan. Locality-IDs as in Table S1.

The second nominal Iranian hynobiid taxon is based on a large, 23 cm long adult male type specimen, deposited in the Muséum National d’Histoire Naturelle in Paris (MNHN). This salamander was discovered in a cave at the eastern edge of the Hyrcanian corridor [10] and only later described as *Batrachuperus gorganensis* Clergue-Gazeau and Thorn, 1979 [11]. Stöck ([12]; therein “Fig. 8”) depicted the type and provided a flow-cytometric DNA measurement (34.77 pg), although based on GC-biased DAPI-staining, see also [13], and a Giemsa-stained karyotype (*2n* = 62), obtained from fin clips of topotypic *Paradactylodon* (as *Batrachuperus*) *gorganensis* larvae (i.e., the eastern taxon). This shows the genome size in the upper part of the range of large amphibian and urodelean genomes [14]. Therefore, whole genome sequencing still remains a major challenge (cf. [15]). Stöck [12] also described external larval morphological changes during the development from a total length of 40 mm until metamorphosis (100 mm). These authors [12] reviewed the specific literature and provided a map with geographic coordinates of all records published until that time ([12]; therein “Fig. 1”). Ebrahimi et al. [16] depicted, measured and compared for the first time the egg sacs of *P. gorganensis* with those of other hynobiid species, showing them to be among the largest of extant hynobiids (surpassed only by eggs sacs of *Ranodon sibiricus* [17,18]). Without providing further taxonomic reasoning, several authors [19,20,21,22,23], published additional data on the biology and distribution of the Iranian hynobiid salamanders from Ardabil and Gilan provinces, all nomenclaturally assigned to *P. persicus*, although several of the specimens were from eastern Iran (i.e., nominal *gorganensis*). Using skeletochronology, a recent paper examined the age structure in topotypic *P. gorganensis* [24] and suggested a lifespan of 13 years for females and 11 years for males.

Based on a complete mitochondrial genome, Zhang et al. [25] showed topotypic *P. gorganensis* to be a ca. 40 My diverged sister taxon of *P. mustersi* from Afghanistan and to form a phylogenetic clade with *Ranodon sibiricus* (see also [26]). While multiple nuclear genes in general supported the age of the clade involving *P. mustersi* and *R. sibiricus* (40 My; [27]), this phylogeny did not include Iranian hynobiids and thus could not further contribute to elucidate their intrageneric or intraspecific relationships.

In the present study, our aims were (i) the clarification of the phylogenetic relationships of the Iranian *Paradactylodon*, (ii) to produce the first genome-wide resource for this rare species (beyond few existing PCR-based sequences); and (iii) to report the first larval and sexual developmental data. To achieve these three goals, we generated phylogenetic data from a geographically comprehensive collection of available DNA-samples from the entire range of Iranian hynobiids, including in part archival museum samples from scientific collections, and study their phylogeny based on three complete mitochondrial genomes. Given the huge genome size of hynobiids that make whole genome analyses still almost unaffordable, as the first genomic resources of *Paradactylodon*, we publish two transcriptomes, based on RNAseq of a whole embryo and a larva. We use these transcriptomes to derive a list of genes involved in sexual development and sex determination in other vertebrates. We also contribute to the sparse knowledge about the ontogenetic development of the species. All of this will facilitate future studies on the genomic level and provides new biological data for these poorly known salamanders.

## 2. Materials and Methods

### 2.1. Animals and Samples

For the present study, we used three (i–iii) sources of DNA-bearing materials (Table S1, Figure 1): (i) tissue samples derived from adults and larvae deposited in museum collections, namely the Natural History Museum of Tehran, Iran (MMTT, Muze-ye Melli-ye Tarikh-i Tabi’i, Tehran), the Bavarian State Collection of Zoology, Munich, Germany (ZSM), and the Senckenberg Collections of Dresden, Germany (MTKD); (ii) mixed tissues of an entire embryo in developmental stage 46 (see below), as staged in *Onychodactylus japonicus* by Iwasawa & Kera [28] or 36–37, as staged in *Hynobius nigrescens* by Iwasawa & Yamashita [29], collected on 24 April 2015 from egg sacs, found at locality 4 (Figure 1) and stored in the field in RNAlater; (iii) multiple tissues from a sibling larva kept in an aquarium and prepared, when reaching a total length of 53 mm at 38 days after hatching, anesthetized by immersion in tricaine methanesulfonate (MS 222; Sigma-Aldrich), transferred into RNAlater and stored at −80 °C (Table 1). This larva presented stage 60 according to Reference [28], stage 57 according to Reference [29], or stage XII of Vassilieva & Smirnov [30].

### 2.2. Gonadal Gross Morphology and Histology

Three selected larvae (Table 1) were anesthetized by immersion in tricaine methanesulfonate (MS 222; Sigma–Aldrich). Whole larvae, with opened peritoneum, were fixed in Bouin’s solution (24 h) and subsequently rinsed several rounds in 70% ethanol. Gonadal anatomy was inspected after removing the digestive tract. Histological sections were prepared and analyses were performed according to established protocols [31,32]. Using Stemi SV11 (Zeiss, Germany) microscope and camera, separated gonads were photographed, embedded in paraplast, sectioned into 7 μm longitudinal slices, stained with Mallory’s trichrome, and examined using a Zeiss Axioskop 20 microscope. Images were acquired by a cooled Carl Zeiss AxioCam HRc CCD camera.

### 2.3. DNA Extraction of Topotypic Samples

We included topotypic tissue from larvae collected by one of us (JFS) on June, 1, 1970 (see Reference [9]), from the type locality of *Paradactylodon persicus* [8] and stored in ethanol, first in a private collection that was meanwhile transferred to ZSM. After unsuccessful experiments in 2004, we extracted DNA from these archival samples using the Qiagen DNeasy Blood & Tissue kit (Qiagen, Hilden, Germany) with modifications, namely including an overnight lysis and 15 min incubation at 37 °C during the elution, in dedicated archival DNA facilities (cf. [33]). For all other tissue samples, extraction followed the manufacturer’s protocols.

### 2.4. Primers Developed for PCR-Amplification of mtDNA-Fragments from Archival Samples

For the majority of the individuals, almost the entire mitochondrial cytochrome *b* was amplified with the newly developed primer pair PgorgCytb_F1/PgorgCytb_R2 (Supplementary Materials Table S2), yielding a ~940 bp product, using the PCR protocol 96 °C, 2 min, initial denaturation, a cycle of 38× including (94 °C, 2 min, denaturation; 53.5 °C, 1 min, annealing; 72 °C, 1.5 min, extension) and final extension at 72 °C for 5 min. For two shorter fragments from archival samples, two primer pairs (PgorgCytb_F1/PgorgCytb_R4 and PgorgCytb_F4/ PgorgCytb_R2; Table S2), amplifying two overlapping pieces, together also spanning the entire cytochrome *b* sequence, were amplified with a slightly varying protocol: 96 °C, 2 min, initial denaturation, 50× cycle including (94 °C, 2 min, denaturation; 53.5 °C, 1 min, annealing; 72 °C, 1 min, extension) and final extension at 72 °C for 5 min. Due to DNA damage, in four topotypic archival DNA samples (ZSM1-ZSM) even shorter fragments of cytochrome *b* had to be amplified, using primers PgorgCytbF2_short/PgorgCytb_R1 under the same conditions.

Samples from good-quality DNA were amplified in a total reaction volume of 25 μL comprising 15.4 μL ddH_2_O, 2.5 μL of 10× Top Taq PCR buffer (Qiagen), 0.47 μL of dNTPs (10 mM each nucleotide), 1.25 μL of each primer, 0.13 μL of Top Taq Polymerase (Qiagen) and 3 μL of DNA (10–30 ng/μL).

For archival samples, the total reaction volume of 20 μL comprised 11.4 μL ddH_2_O, 4 μL of 5× Phusion PCR buffer, 0.4 μL of dNTPs (10 mM each nucleotide), 1 μL of each primer, 0.2 μL (2 U/rxn.) of Phusion Hot Start II High-Fidelity DNA Polymerase (Thermo Fisher Scientific) and 2 μL of DNA (10–30 ng/μL). In all cases, PCR success was tested on 1.5% agarose gels using 4 μL of product; the remaining volume was used for Sanger sequencing in one or both directions, depending on product size.

### 2.5. Sequencing and Reconstruction of a Complete mtDNA-Genome of Topotypic *Paradactylodon persicus*

Of one topotypic DNA archival sample (ZSM 1, loc. 1), of which small fragments (ca. 140 bp) of mtDNA had been successfully PCR-amplified as described above, we constructed two libraries from two independent DNA extracts, following [34], incorporating 8-nt barcodes into both adapters. Libraries were then sequenced on the Illumina NextSeq 500 (Illumina Inc.)., using a mid-output 150-cycle kit at the Berlin Center for Genomics in Biodiversity Research (BeGenDiv). Paired end-reads were demultiplexed with bcl2fastq v2.17.1.14 (Illumina Inc.). CUTADAPT v1.3 [35] was used to remove Illumina adapters, Trimmomatic [36] for quality-trimming in a sliding window approach, and sequences were merged (Flash v1.2.; [37]). Sequences were aligned to the reference mitogenome (*P. gorganensis*, GenBank accession no. NC_008091.1 [25]) using BWA v0.7.10 [38], and duplicate sequences were removed using MarkDupsByStartEnd.jar (https://github.com/dariober/Java-cafe/tree/master/MarkDupsByStartEnd). The consensus sequence was then generated based on a coverage threshold of ≥3×. A second, almost complete mitochondrial genome (16,057 bp) was assembled using the same bioinformatics pipeline from the larval transcriptome, obtained at locality 4.

### 2.6. Phylogenetic Analyses

**Figure 2:**
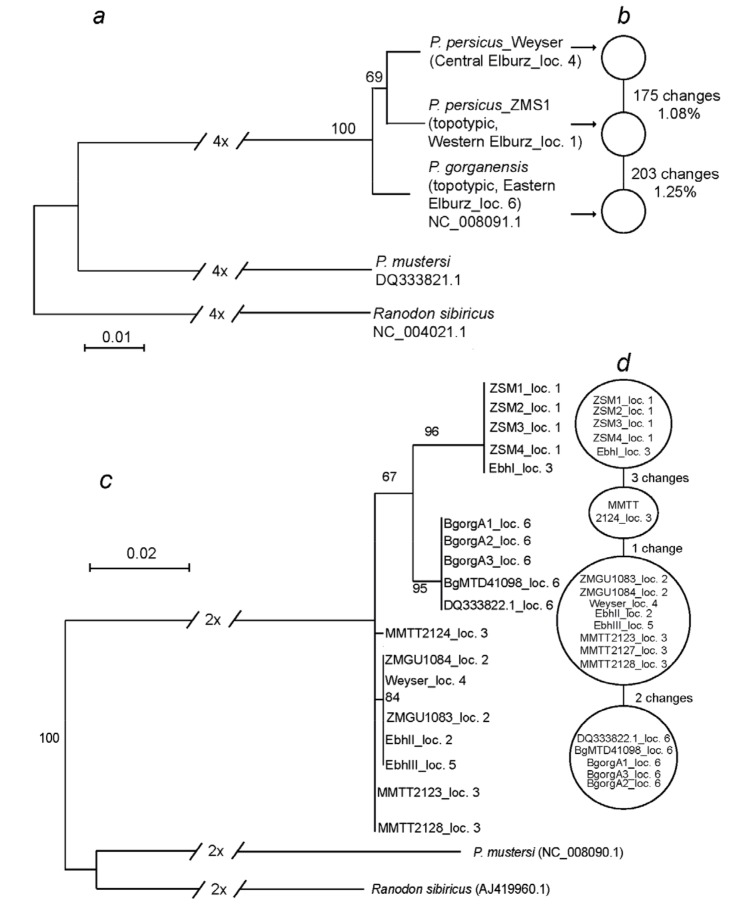
Phylogeny of Central Asian and Iranian hynobiid salamanders and network of Iranian *Paradactylodon*. (**a**) Maximum-likelihood phylogeny with branch support from 100 bootstrap pseudoreplicates using PhyML in % based on almost complete mitochondrial genomes (16,300 bp), (small arrows in **a** refer to the corresponding sample names in **b**; (**b**) Parsimony-based network of the three mtDNA genomes (16,140 bp). (**c**) Maximum likelihood phylogeny based on 865 bp of the cytochrome *b* fragments, parameters as in **a**; (**d**) Parsimony-based network of the 119 bp of short fragments obtained by PCR of archival samples. For sample numbers and localities: Figure 1 and Table S1. Note that samples labeled ZSM1-ZSM4 all belong to larvae, jointly archived under collection label ZSM 821/2006 (Table S1); bootstrap support values are only shown when >65. According to the present paper, all ingroup-sequences belong to a single species, *Paradactylodon persicus* sensu lato (= s.l., see Discussion).

Mitochondrial DNAs of *P. persicus* from the type locality near Assalem (loc. 1) and from central Elburz (loc. 4) were aligned with that of topotypic *P. gorganensis* (GenBank NC_008091.1) from eastern Elburz (loc. 6); *P. mustersi* (GenBank DQ333821.1) and *Ranodon sibiricus* (GenBank NC_004021.1) were used as outgroups. To infer the best fitting model of sequence evolution (GTR+G, gamma-shaped: 0.197; Akaike criterion), we used SMS [39]. To illuminate the intraspecific phylogeny of Iranian *Paradactylodon*, we ran PhyML [40] with this inferred substitution model and 100 bootstrap pseudoreplicates (Figure 2a). In addition, to better evaluate and depict the relationships and total differences between the three mitochondrial genomes from Iran, we used PopART (Population Analysis with Reticulate Trees; http://popart.otago.ac.nz/index.shtml), an open source population genetics software, developed by the Allan Wilson Centre Imaging Evolution Initiative. We removed sites with missing data that could not be obtained from archival specimens and created ancestral parsimony-based networks. To analyze the mtDNA-phylogeography throughout the range (locs. 1–6), we used an alignment of up to 865 bp of the mitochondrial cytochrome *b*, that was supplemented with all available fragments obtained by PCRs as described from the archival specimens and analyzed them with the same software and under the maximum likelihood parameters as above and added a parsimony-based network based on only 119 bp, available from all samples.

### 2.7. Sequencing of Two Transcriptomes, Functional Annotation, and Classification

RNA was extracted using a standard Trizol protocol from mixed tissues from the whole embryo (after removing the yolk sac) and from a larva (Table 1), using single organs (liver, eye, brain, heart, muscle, and gonad). Larval organs’ RNAs were adjusted to equal concentrations and pooled before RNAseq. Complementary DNA (cDNA) was synthesized and sequenced by BGI (BGI-Hongkong Co., Ltd., China), using the Hiseq4000 sequencing system (Illumina, San Diego, CA, USA). Reads were assembled using Trinity [41]; Tgicl [42] was used to assemble transcripts to unigenes. For annotation, BLASTx (v2.2.23; [43]) was used to align unigenes to five protein databases (NT, NR, COG, KEGG, and SwissProt), and Blast2GO [44] to obtain GO annotations.

### 2.8. Genes Involved in Sex Determination or Sex Differentiation

We aimed at retrieving 37 genes potentially involved in male or female sex determination and sexual differentiation (list by M. Schartl, pers. comm., modified for Urodela). Template protein sequences from the anuran model species *Xenopus tropicalis* or *X. laevis* were obtained from Xenbase [45] and aligned with BLAT [46] (−t = dnax −q = prot) against *P. persicus* transcripts, assembled from RNAseq data. Matching transcripts were extracted, aligned against the NR database using NCBI blastx [43], and individually inspected to identify and remove paralogs or unspecific matches. Transcripts were considered homologous, if the vast majority of the top scoring BLASTx hits were related to the gene of interest.

Genes from the list that were not found in the transcriptome assemblies, were aligned with the raw RNAseq read data, using a local BLAST approach (45,774,124 sequences; 4,577,412,400 total nucleotides). Here, available template RNA or protein sequences were used as queries for tblastn or tblastx approaches, preferentially of Urodela (if available) or Anura. Reads with matches were extracted and assembled by the IDBA transcriptome assembler [47]. Assembled transcripts were checked by NCBI BLASTx as above. If no transcripts for the gene of interest could be successfully assembled, the extracted reads were first aligned with the *P. persicus* transcriptome assembly (BLAT default parameters) to sort out paralogous gene family matches. Subsequently, reads not already matching other *Paradactylodon* paralogs were submitted to NCBI BLASTx and manual inspection to reveal low-expression-level transcripts.

## 3. Results

### 3.1. Transcriptomes

In total, we have generated approximately 9.17 Gb bases of RNA sequences by Illumina Hiseq. Assembly of all samples yielded 78,918 unigenes, with a total length of 77,071,006 bp, an average length of 976 bp, an N50 value of 2019 bp (Figure S1), and a GC content of 46.49%. Raw reads from both transcriptomes were deposited in the NCBI Sequence Read Archives (SRA, http://www.ncbi.nlm.nih.gov/Traces/sra/; SUB3150164, SUB3166015) under the BioSample accession number PRJNA415277. Annotation of unigenes in seven functional databases yielded 35,780 (NR: 45.34%), 29,271 (NT: 37.09%), 30,414 (Swissprot: 38.54%), 10,920 (COG: 13.84%), 28,732 (KEGG: 36.41%), 7388 (GO: 9.36%), and 25,944 (Interpro: 32.87%) of annotated unigenes (File S1). Using functional annotation results (Figure S2), we detected 35,888 CDS (coding sequences), and after gene prediction using ESTScan [48] on the remaining unigenes, we obtained 3346 additional CDS. We have also detected 11,337 SSRs (simple sequence repeats; micro- and mini-satellites), distributed on 8802 unigenes.

### 3.2. Genes Involved in Sex Determination or Sex Differentiation

Of a list 37 genes (Table S3), ca. 86% (26) had significant blast hits in the *P. persicus* transcriptomes in *Xenopus*, providing the first sequence data for these genes for this rare urodelean species or even the family Hynobiidae. For six other important vertebrate genes involved in sex determination or sex differentiation (DMRT1, DMRT3, AMHR2, FOXL2, SF1, and WNT1), at least a few reads yielded Blast hits when urodelean (or other amphibian) queries were used (Table S3, File S2). However, no matches were obtained for the five remaining genes (ALDH1A2, DMRT6/DMRTB1, FGF16, NR0B1, and SRD5A3).

### 3.3. Mitochondrial Genomes

The two sequencing libraries of sample ZSM 1 from topotypic *P. persicus* yielded combined 7,704,874 PE-reads of archival DNA. In total, only 2511 sequences mapped to the reference mitogenome (i.e., only 0.03% of the shotgun data), covering 16,140 bp of assembled mtDNA. In addition, from one transcriptome (loc. 4), we generated a total of 16,057 bp of mitochondrial DNA. Sequences are deposited in GenBank (accession numbers MK737945-MK737946).

### 3.4. Phylogenetic Analyses

In the maximum likelihood phylogeny, the three Iranian *Paradactylodon* mtDNA-genomes (Figure 2a) formed a highly supported clade, with the mtDNAs from the Western (loc. 1) and central Elburz (loc. 4) falling into a very weakly supported subclade, as compared to the topotypic, nominal *P. gorganensis* (loc. 6). Accordingly, the network (based on 16,140 bp, equally available from all three Iranian *Paradactylodon* mtDNAs; Figure 2b) shows 175 changes between *Paradactylodon* from localities 1 and 4 (ca. 1.08%) but 203 (ca. 1.25%) between those from localities 1 and 6. Additional phylogenetic analyses of fragments of up to 865 bp of the mitochondrial cytochrome *b*, obtained with overlapping PCRs from many more, in part archival samples (Figure 2c), show a slightly differing topology (most probably due to missing data from some samples) but clearly confirm close relationships between all *P. persicus* sensu lato (i.e., comprising nominal *P. persicus* as well as *P. gorganensis*), as does a network, constructed from only 119 bp of this gene, equally available from each of these samples (Figure 2d).

### 3.5. Larval Ontogeny with First Observations of Gonadal Development

**Figure 3:**
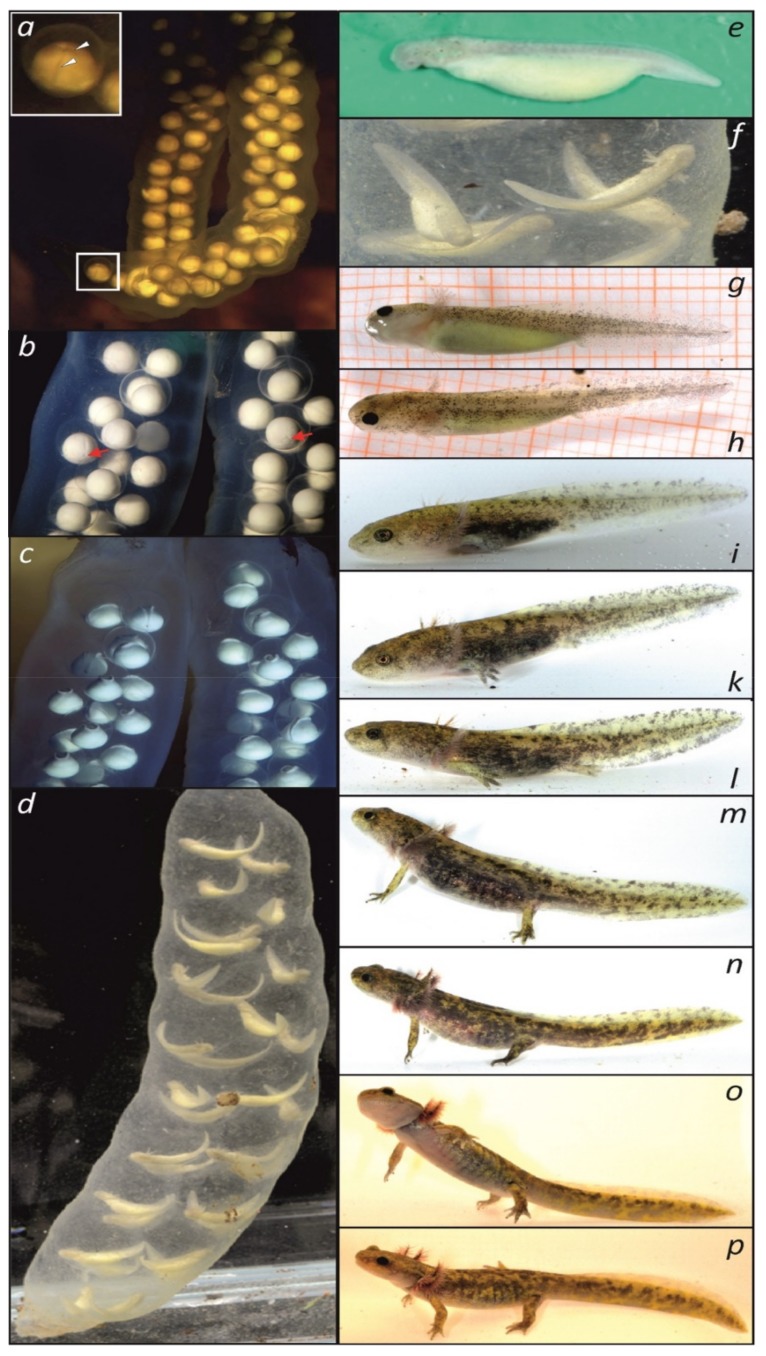
Development of *Paradactylodon persicus* (for staging see Table 1). (**a**–**c**) Egg sacs of descendants of *Paradactylodon* (topotypic *P. gorganensis*), obtained at loc. 6 that reproduced in captivity at 6.5 °C water temperature. (**a**) day 1 after spawning, visible cleavage (white arrow head); (**b**) detail of the sac with gastrulation visible (red arrow), 12 days after spawning; ***c*:** neurulation at day 18 after spawning; (**d−p**) developmental series of *P. persicus* in captivity over 58 days, during which water temperature raised from 12–20°C; at each stage, the largest larva is shown (**d**) one of two egg sacs (photographed in a cuvette) found at locality 4 in 1550 m a.s.l. at 8–9 °C water temperature; (**e**) isolated single embryo (about 2 cm) from the sac shown in (**d**), of which one transcriptome was sequenced, after the yolk was removed; (**f**) detail of *d* with external gills and yolk sacs of embryos visible; (**g**) and (**h**) larvae at day 4 after hatching (lasting 14 days from the same egg sac at 12 °C; gonadal histology in Figure 4a–c); (**i**) 24 days (gonadal histology in Figure 4d–f); ***k***: 29 days; (**l**) 38 days: larval stage at which RNA of six organs was obtained for the second transcriptome.; (**m**) 41 days (gonadal histology in Figure 4g–i); (**n−p**) three different larvae at 58 days; salamanders left water at ca. 60 days at 60 mm total length. **Photographs**: (**a**–**c**) Michael Fahrbach, (**d**–**p)** Sebastian Voitel.

**Figure 4:**
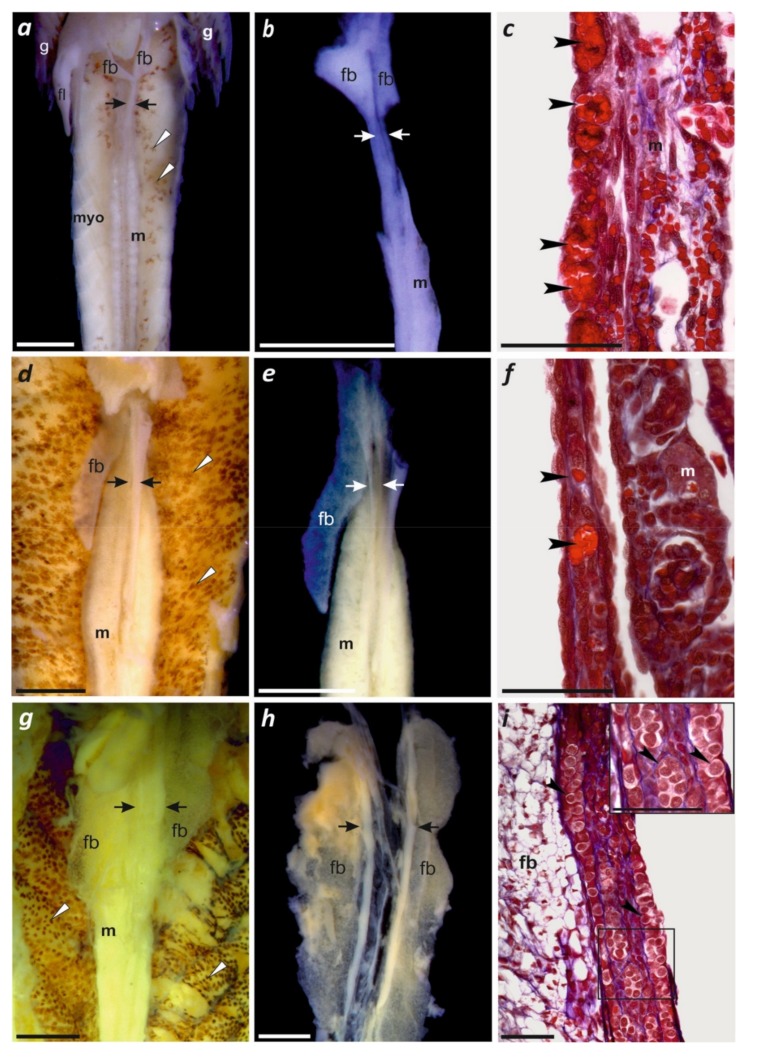
Developing gonads of *Paradactylodon persicus* (for staging: Figure 3 and Table 1). (**a**–**c**) Larva at day 4 after hatching; (**d**–**f**) at day 24 after hatching; (**g**–**i**) at day 41 after hatching; (**a**), (**d**) and (**g**) *situs viscerum* (digestive tract removed). Note the position of gonads (outlined in black: Figure S3) in relation to the mesonephroi; (**b**), (**e**) and (**h**) gonads dissected with fat bodies; (**c**) and (**f**) longitudinal section of undifferentiated gonads containing primordial germ cells loaded with yolk platelets (stained red); (**i**) fat body and gonocytes in early testis; inset: enlarged portion of the testis core invaded by a group of gonocytes. **Abbreviations: fb**: fat body**; fl**: forelimb bud; **g**: gills; **m**: mesonephros; **myo**: trunk musculature (myomeres separated by myoseptae); white arrowheads: melanophores in the dorsal peritoneum; black arrowheads: germ cells; white or black arrows: gonads. **Scale bars**: a, b, d, e, g, h: 1 mm; c, f, i: 100 µm.

We present a photographic sequence of the ontogenetic development from the first day of spawning, first cleavage, formation of the neural crest, advanced embryos, and then from hatching until metamorphosis (Figure 3), which allowed us to compare stages with other hynobiids (Table 1) and to assign these stages to the early gonadal development of larval *P. persicus* for the first time. The gonads are paired elongated organs, situated longitudinally in the medial line of the body, parallel to the proximal portions of the mesonephroi (Figure 4a,d,g; see also Figure S3a,d,g). Until day 29 after hatching, the gonads remained sexually undifferentiated and contain big primordial germ cells (PGCs), loaded with yolk (Figure 4c,f). At day 41 after hatching, the gonads started sexual differentiation (Figure 4g–i). Fat bodies, which differentiated from the most proximal portions of the gonadal anlage (Figure 4b), further developed towards the caudal part of the body (Figure 4), in parallel to the gonads and up to their distal tips (Figure 4h). At the time of sexual differentiation, their cells were filled with fat droplets (Figure 4i).

## 4. Discussion

### 4.1. Transcriptomes of Embryo and Larva of Paradactylodon Among the First in Hynobiidae

Mainly due to enormous genome size [14], amphibian genomics, and particularly that in urodela, remains extremely challenging, with only one model urodelan species’ genome fully sequenced [15], but even there is awaiting improved annotation. The genome size of *Paradactylodon* (ca. 34.7 pg/nucleus; [12]) probably keeps whole genome sequencing unaffordable in the near future. Therefore, beyond a limited number of existing PCR-based sequences, the two transcriptomes provide a first genome-wide resource for the genus and to our knowledge the second and third in Hynobiidae [49]. The first transcriptome in Hynobiidae was studied *Hynobius chinensis* [49], a taxon, which is about 55 My diverged from *Paradactylodon* (www.timetree.org; average of eight molecular studies), and thus expected to show major evolutionary differences.

Our research revealed the stage of sexual differentiation of gonads in *P. persicus* (histologically documented at day 41), and accordingly, in the second larval transcriptome, prepared at day 38 after hatching (Table 1), we detected low levels of expression of candidate genes that may be especially relevant for sex determination (Table S3, such as DMRT1, AMH, AMHR2, and FOXL2. This might facilitate future studies in the genus *Paradactylodon* and other Hynobiidae. Sex chromosomes of most salamanders are homomorphic [50,51], and in most species, mainly the observation of balanced sex ratios from clutches is interpreted as indication for genetic sex determination but has remained essentially without genetic evidence [52]. In Hynobiidae, gene expression in context to sexual development has been studied using histology and qPCR of a single gene (P450) in *Hynobius retardus* by Sakata et al. [53]. They have shown that P450 aromatase was expressed predominantly in the adult ovary and brain, weakly in testis, but not in other somatic organs. A typical sexual dimorphism in P450 aromatase expression was detected in normally developing larvae by a quantitative competitive RT-PCR; strong expression in the female gonads but very weak in male gonads [53].

### 4.2. Gonadal Differentiation in Paradactylodon and Other Hynobiid Salamanders

Morphological and histological data of adult Hynobiidae have been reported to asses the reproductive cycles in both sexes of *Salamandrella schrenckii* [54] and *S. keyserlingii* [54,55,56,57,58]. The larval development of gonads from 0–60 days was described in *Hynobius retardatus* [50] at 16–20 °C. In this species, genital ridges with primordial germ cells (PGCs) were formed within 10 days after hatching and sexual differentiation occurred within 20–30 days after hatching (stage 53 of [29]; Table 1).

Here, we document the earliest stages of gonad differentiation in *Paradactylodon*, just after PGCs had invaded the gonadal anlage in stages 42–43 and 55 (at day 24 and 29 after hatching, Table 1). PGCs were clearly distinguishable by their big size and heavy yolk load, as in other amphibians (for review: [59]). Sexual differentiation of gonads–in this case testes–took place before metamorphosis, at stage 63, at day 41 of (hatched) larval life. The differences in time (20–30 days) required to achieve sexual differentiation reported for *H. retardatus* [53] and *P. persicus* (41 days, this study) seemed to be not only species-specific, but also resulted from rearing temperature (16–20 °C and 12–20 °C, respectively). Development of gonads in *H. retardatus* during 1–3 years of its life was described by Kanki and Wakahara [60]. Unfortunately, similar later stages of gonad development were not available in our study.

### 4.3. Phylogeography, Taxonomy, and Conservation

Using three almost complete mitochondrial genomes from the three geographic extremes, involving both type localities (locs. 1, 6) and a sample from central Elburz (loc. 4), we were able to perform a comparative phylogenetic analysis of Iranian *Paradactylodon*, supplemented with additional archival samples and available sequences from GenBank (Figure 2a–d). The largest genetic distance between these three mitochondrial genomes is about 2%, suggesting that these secretive salamanders may belong to a single species, however, clearly exhibiting some intraspecific variation. While our data suggest that both taxa belong to a single species, we also can see some variation along the Hyrcanian Forests of Iran, suggesting there is “clinal variation” along their range corridor as previously proposed [12]. The distances reported among the *Paradactylodon* mitochondrial DNAs (1–2%, Figure 2) roughly correspond to those between “subspecies” or within species in some other Urodela (e.g., Plethodontidae [61,62] and Salamandridae [63,64]), including Hynobiidae. Using COI and 16S rDNA, Xia et al. [65] found 2-parameter genetic distances (K2P) of the mean intraspecific variation for COI and 16S rDNA to be 1.4% and 0.3%, respectively. Uncorrected pairwise distances of cytochrome *b* in multiple *Batrachuperus* species from East Tibet were found to show even larger intraspecific variation [66].

So far, according to the International Union for Conservation of Nature (IUCN) red list, these two nominal species of Iranian hynobiids are considered “critically endangered” (*P. gorganensis*) or “near threatened” (*P. persicus*). Although our data on mitochondrial DNA suggest that they presumably represent a single species, further studies on nuclear genes across the entire distributional area are required. Hence, we plead for strict conservation of endangered populations of “*P. persicus* sensu lato” throughout their range.

The nomenclature of the genus *Paradactylodon* has been debated for many years [12,67,68]. The genus has recently also been called *Iranodon*, however, *Paradactylodon* Risch 1984 was in “error considered a *nomen nudum* by Dubois and Raffaëlli, 2012” [69], according to Frost [70].

These salamanders can be considered as bioindicators for “intact and healthy” stream ecosystems of the Hyrcanian Forests. However, in some cases, *P. persicus* might persist for decades even after anthropogenic deforestation has long destroyed the native vegetation [21], as long as intact streams or springs allow relic populations to endure. These hynobiids have survived in the Hyrcanian Forests since the Tertiary; therefore, humanity and namely Iran has a great responsibility to protect these species within their ecosystems in the long term, along with the unique biodiversity of their habitats [71].

## Figures and Tables

**Table 1:** Developmental stages and samples taken for transcriptomics and gonadal histology of *Paradactylodon* in comparison to stages in three genera of hynobiid salamanders (*Onychodactylus*, *Hynobius*, and *Ranodon*), according to different authors.

Developmental Stages in					
*Paradactylodon persicus*			*Onychodactylus japonicus* (Iwasawa & Kera 1980) [28]	*Hynobius nigrescens* (Iwasawa & Yamashita 1991) [29]	*Ranodon sibiricus* (Vassilieva & Smirnov 2001 [30]
Photographs Figure 3	Days of Development	Samples Examined			
*a*	1 day after spawning embryos in the sac, cleavage	–	3–4	3	–
*b*	12 days after spawning, embryos in the sac, gastrulation	–	22	12B	–
*c*	18 days after spawning, embryos in the sac, neural plates	–	29	18	–
*e*	single embryo, egg sac removed	Transcriptome 1	46	36–37	–
*f*	larvae before hatching, external gills	–	48	39	–
*g, h*	larva at day 4 after hatching	Gonadal histology (Figure 4a–c)	49	42–43	II
*i*	24 days	–	55	53	VI
*k*	29 days	Gonadal histology(Figure 4d–f)	58	55	VII–IX
*l*	38 days	Transcriptome 2	60	57	XII
*m*	41 days	Gonadal histology(Figure 4g–i)	66	63	XV
*n*	58 days	–	69	65	–
*o*	58 days	–	69	65	–
*p*	60 days	–	69	66	–

## Supplementary Materials

Supplementary materials can be found at https://www.mdpi.com/2073-4425/10/4/306/s1. Table S1. List of samples of Iranian *Paradactylodon*; sample IDs according to Figure 1 (PDF). Table S2. Primers used to amplify mtDNA fragments of cytochrome *b* of *Paradactylodon* (PDF). Table S3. Male and female sex-determining and sex differentiation gene inventory and homologous *P. persicus* transcripts (red entries refer to low levels of expression) (PDF). Figure S1. Frequency length distribution of unigenes in the *Paradactylodon persicus* transcriptome. (JPG). Figure S2. Functional GO annotations of *Paradactylodon persicus* transcriptome unigenes. (JPG). Figure S3. Annotated version of Figure 4 with the gonads outlined (JPG). File S1. Annotation results of unigenes from five databases (txt). File S2. Contigs of RNA sequences of extracted male and female sex-determining and sex differentiation genes, obtained from the *P. persicus* transcriptomes (txt).
