# Supplementary material for: Shedding Light on a Secretive Tertiary Urodelean Relict: Hynobiid Salamanders (Paradactylodon persicus s.l.) from Iran, Illuminated by Phylogeographic, Developmental, and Transcriptomic Data
Source: Genes (Basel). 2019 Apr 18;10(4):306. doi: 10.3390/genes10040306 (PMC6523714; doi:10.3390/genes10040306)
Supplement: Supplementary file 1 [file genes-10-00306-s001.zip › SupplementaryMaterials_StoeckEtAl/TableS3_StoeckEtAl.pdf]

Proteins used for screening					Matches in Transcriptome		Top BLASTX result for matches against NCBI NR database						
Accession	Organism	Gene	Length of Protein (aa)	Length of mRNA (nt)	*Paradactylodon persicus*, transcript ID	Transcript length [bp]	Description	Max score	Total score	Query cover	E value	Ident	Accession
XP_002939310.1	X. tropicalis	**ALDH1A3**	512	4056	**Unigene66003_All**	581	PREDICTED: aldehyde dehydrogenase family 1 member A3-like [Xenopus laevis]	361	361	99,00%	7,00E-124	88,00%	XP_018110759.1
XP_002939310.1	X. tropicalis	**ALDH1A3**	512	4056	**Unigene66004_All**	534	PREDICTED: aldehyde dehydrogenase family 1 member A3 isoform X1 [Nanorana parkeri]	283	283	99,00%	2,00E-93	76,00%	XP_018415754.1
XP_002939310.1	X. tropicalis	**ALDH1A3**	512	4056	**Unigene65842_All**	202	PREDICTED: aldehyde dehydrogenase family 1 member A3 isoform X2 [Nanorana parkeri]	122	122	98,00%	2,00E-34	89,00%	XP_018415755.1
XP_002939310.1	X. tropicalis	**ALDH1A3**	512	4056	**Unigene43849_All**	337	PREDICTED: aldehyde dehydrogenase family 1 member A3 [Xenopus tropicalis]	141	141	64,00%	6,00E-40	85,00%	XP_002939310.1
XP_002941888.2	X. tropicalis	**AR**	788	3497	**Unigene49431_All**	2584	PREDICTED: androgen receptor [Xenopus tropicalis]	841	841	77,00%	0	61,00%	XP_002941888.2
NP_001016958.1	X. tropicalis	**CTNNB1**	781	3382	**Unigene1104_All**	3258	catenin beta [Xenopus laevis]	1545	1545	71,00%	0	97,00%	NP_001084045.1
NP_001080681.1	X. laevis	**CXCR4B**	358	2115	**Unigene4849_All**	1851	C-X-C chemokine receptor type 4-B [Xenopus laevis]	518	518	54,00%	0	79,00%	NP_001080681.1
AAI71087.1	X. tropicalis	**CYP26A1**	492	1458	**Unigene34218_All**	417	cytochrome P450 Cyp26a1 [Andrias davidianus]	283	283	99,00%	2,00E-94	97,00%	ARO89874.1
AAI71087.1	X. tropicalis	**CYP26A1**	492	1458	**Unigene7192_All**	1097	cytochrome P450 Cyp26a1 [Andrias davidianus]	635	635	88,00%	0	94,00%	ARO89874.1
AAI35552.1	X. tropicalis	**CYP26B1**	511	6137	**Unigene41846_All**	338	cytochrome P450 family 26 subfamily B polypeptide 1 [Xenopus laevis]	219	219	99,00%	9,00E-71	96,00%	ACF33501.1
AAI35552.1	X. tropicalis	**CYP26B1**	511	6137	**Unigene59057_All**	314	cytochrome P450 26B1 [Xenopus tropicalis]	130	130	92,00%	2,00E-36	93,00%	NP_001072655.1
AAI35552.1	X. tropicalis	**CYP26B1**	511	6137	**Unigene16612_All**	2387	cytochrome P450 26B1 [Xenopus tropicalis]	438	438	36,00%	6,00E-145	91,00%	NP_001072655.1
XP_002939137.2	X.tropicalis	**CYP26C1**	533	4692	**Unigene29794_All**	2340	PREDICTED: cytochrome P450 26C1 [Nanorana parkeri]	714	714	75,00%	0	65,00%	XP_018414201.1
NP_001083086.1	X. laevis	**ESR1A**	585	-	**Unigene43178_All**	249	estrogen receptor alpha [Hynobius tokyoensis]	166	166	100,00%	1,00E-49	99,00%	BAJ05027.1
NP_001083086.1	X. laevis	**ESR1A**	585	-	**Unigene65458_All**	434	estrogen receptor alpha [Cynops pyrrhogaster]	273	273	99,00%	2,00E-89	97,00%	BAJ05025.1
ACK58680.1	X. tropicalis	**FGF20**	208	1490	**Unigene49152_All**	208	fibroblast growth factor 20 L homeolog [Xenopus laevis]	142	142	99,00%	5,00E-44	93,00%	NP_001083766.1
NP_001016949.1	X. tropicalis	**GATA-4**	394	1599	**Unigene35647_All**	314	GATA binding protein 4 [Notophthalmus viridescens]	138	138	99,00%	6,00E-42	88,00%	ADO14130.1
NP_001016949.1	X. tropicalis	**GATA-4**	394	1599	**Unigene35648_All**	206	PREDICTED: transcription factor GATA-4 isoform X1 [Xenopus tropicalis]	147	196	99,00%	4,00E-44	99,00%	XP_012819495.1
NP_001016949.1	X. tropicalis	**GATA-4**	394	1599	**Unigene11415_All**	320	GATA binding protein 4 [Notophthalmus viridescens]	66,6	66,6	42,00%	7,00E-14	64,00%	ADO14130.1
NP_001039203.1	X. tropicalis	**LRPPRC**	1391	4347	**Unigene13522_All**	4523	PREDICTED: leucine-rich PPR motif-containing protein, mitochondrial [Xenopus laevis]	1308	1910	89,00%	0	68,00%	XP_018118039.1
AAI60575.1	X. tropicalis	**PDGFb**	240	2140	**Unigene48562_All**	221	PREDICTED: platelet-derived growth factor subunit B [Nanorana parkeri]	87,4	87,4	99,00%	6,00E-22	63,00%	XP_018425766.1
XP_002937129.2	X. tropicalis	**PTCH2**	1423	6786	**CL1064.Contig1_All**	907	PREDICTED: protein patched homolog 2 isoform X2 [Xenopus tropicalis]	485	485	99,00%	2,00E-161	87,00%	XP_017949029.1
XP_002937129.2	X. tropicalis	**PTCH2**	1423	6786	**CL1064.Contig2_All**	368	RecName: Full=Protein patched homolog 2; Short=PTC2	248	248	99,00%	4,00E-84	94,00%	O42334.1
XP_002937129.2	X. tropicalis	**PTCH2**	1423	6786	**Unigene18572_All**	400	patched 2 S homeolog [Xenopus laevis]	212	212	99,00%	1,00E-63	85,00%	NP_001081438.1
XP_002937129.2	X. tropicalis	**PTCH2**	1423	6786	**Unigene34432_All**	499	patched 2 S homeolog [Xenopus laevis]	292	292	99,00%	3,00E-91	80,00%	NP_001081438.1
XP_002937129.2	X. tropicalis	**PTCH2**	1423	6786	**Unigene37507_All**	276	patched 2 S homeolog [Xenopus laevis]	176	176	98,00%	7,00E-52	89,00%	NP_001081438.1
XP_002937129.2	X. tropicalis	**PTCH2**	1423	6786	**Unigene18041_All**	1438	PREDICTED: protein patched homolog 2 isoform X2 [Xenopus tropicalis]	436	436	90,00%	4,00E-140	66,00%	XP_017949029.1
NP_001121500.1	X. tropicalis	**RSPO-1**	257	1946	**Unigene34747_All**	224	PREDICTED: R-spondin-1-like [Xenopus laevis]	112	112	97,00%	1,00E-31	74,00%	XP_018102205.1
NP_001093691.1	X. tropicalis	**SOX10**	436	2895	**Unigene12569_All**	898	Sry-type HMG box 10 [Ambystoma mexicanum]	384	384	72,00%	5,00E-132	86,00%	ABI97016.1
NP_001093691.1	X. tropicalis	**SOX10**	436	2895	**Unigene22036_All**	1852	Sry-type HMG box 10 [Ambystoma mexicanum]	304	304	37,00%	5,00E-96	77,00%	ABI97016.1
XP_002932315.2	X. tropicalis	**SOX8**	466	2389	**Unigene30193_All**	227	PREDICTED: transcription factor Sox-8-like [Xenopus laevis]	111	111	91,00%	1,00E-29	75,00%	XP_018094788.1
XP_002932315.2	X. tropicalis	**SOX8**	466	2389	**Unigene248_All**	1596	transcription factor Sox-8 [Xenopus laevis]	469	469	64,00%	1,00E-161	82,00%	NP_001083964.1
AAT72000.1	X. tropicalis	**SOX9**	482	2538	**Unigene22134_All**	1033	Sox9 [Andrias davidianus]	405	405	66,00%	2,00E-142	96,00%	ADK60865.1
AAT72000.1	X. tropicalis	**SOX9**	482	2538	**Unigene14974_All**	2187	SOX9 [Pleurodeles waltl]	379	379	32,00%	4,00E-123	87,00%	ACF95883.1
NP_001006841.1	X. tropicalis	**SRD5A1**	257	1537	**Unigene6891_All**	1170	PREDICTED: 3-oxo-5-alpha-steroid 4-dehydrogenase 1 [Nanorana parkeri]	311	311	64,00%	1,00E-104	64,00%	XP_018421826.1
NP_001239015.1	X. tropicalis	**WNT4**	351	1962	**Unigene63568_All**	324	WNT family member 4 [Ambystoma mexicanum]	221	221	99,00%	2,00E-72	97,00%	ASW28001.1
NP_001135625.1	X. tropicalis	**WT1**	413	6193	**Unigene326_All**	2082	Wilms tumor protein 1 [Andrias davidianus]	773	773	56,00%	0	90,00%	ANA12157.1
AGC60000.1	P. waltl	**AMH**	336	1008	**AMH_part1**	331	anti-Mullerian hormone [Pleurodeles waltl]	129	129	92,00%	5,00E-34	57.84%	AGC60000.1
AGC60000.1	P. waltl	**AMH**	336	1008	**Unigene51761_All**	260	anti-Mullerian hormone [Pleurodeles waltl]	69,7	69,7	81,00%	6,00E-12	64.38%	AGC60000.1
NM_001007190.1	X. tropicalis	**HHIP**	669	2717	**HHIP_partial**	527	PREDICTED: hedgehog-interacting protein [Gavialis gangeticus]	303	303	93,00%	7,00E-98	86.06%	XP_019377383.1
NM_001007190.1	X. tropicalis	**HHIP**	669	2717	**Unigene12393_All**	3251	HHIP-like protein 1 [Terrapene mexicana triunguis]	1283	1283	74,00%	0	75.97%	XP_024070444.2
NM_001170497.1	X. tropicalis	**PDGFa**	660	1574	**Unigene17455_All**	1817	PREDICTED: platelet-derived growth factor subunit A isoform X2 [Alligator mississippiensis]	336	336	32,00%	2,00E-109	81.63%	XP_006272417.1
NM_001097169.1	X. tropicalis	**DHH**	396	4372	**DHH_partial**	395	desert hedgehog [Pelodiscus sinensis]	220	220	98,00%	4,00E-71	81.54%	BAE78807.1
ANB78803.1	A. mexicanum	**FGF9**	155	465	**FGF9_partial**	407	fibroblast growth factor 9 [Ambystoma mexicanum]	268	268	99,00%	3,00E-90	95.56%	ANB78803.1
NM_001017113.2	X. tropicalis	**SRD5A2**	239	1730	**SRD5A2_partial**	698	PREDICTED: 3-oxo-5-alpha-steroid 4-dehydrogenase 2 isoform X1 [Xenopus tropicalis]	302	302	76,00%	7,00E-101	76.54%	XP_017949395.1
ABB83370.1	P. waltl	**DMRT1**	377	1131	few reads only	2 * 100 bp	doublesex- and Mab-3-related transcription factor 1 [Pleurodeles waltl]	45,4	45,4	72,00%	4,00E-04	79.17%	ABB83370.1
XM_018559472.1	N. parkeri	**DMRT3**	449	1550	few reads only	2 * 100 bp	doublesex- and mab-3-related transcription factor 3 [Xiphophorus couchianus]	44,7	44,7	78,00%	5,00E-04	80.77%	XP_027889369.1
XM_018091717.1	X. tropicalis	**AMHR2**	389	3860	few reads only	2 * 100 bp	anti-Muellerian hormone-receptor 2 [Latimeria menadoensis]	50,8	50,8	99,00%	3,00E-06	69.70%	CCP19125.1
KU360261.1	A. davidianus	**FOXL2**	420	1709	few reads only	2 * 100 bp	forkhead box protein L2 [Chrysemys picta bellii]	56,6	56,6	99,00%	3,00E-06	78.79%	XP_005282573.1
KM215738.1	A. davidianus	**SF1**	496	2098	few reads only	2 * 100 bp	SF1 [Andrias davidianus]	63,5	63,5	96,00%	1,00E-10	87.50%	AIU44171.1
X55270.1	A. mexicanum	**WNT1**	369	2680	few reads only	2 * 100 bp	proto-oncogene Wnt-1 [Apteryx rowi]	76,6	76,6	99,00%	2,00E-16	96.97%	XP_025911231.1
