# Supplementary material for: Shedding Light on a Secretive Tertiary Urodelean Relict: Hynobiid Salamanders (Paradactylodon persicus s.l.) from Iran, Illuminated by Phylogeographic, Developmental, and Transcriptomic Data
Source: Genes (Basel). 2019 Apr 18;10(4):306. doi: 10.3390/genes10040306 (PMC6523714; doi:10.3390/genes10040306)
Supplement: Supplementary file 1 [file genes-10-00306-s001.zip › SupplementaryMaterials_StoeckEtAl/TableS2_StoeckEtAl.pdf]

Supplementary Table S2. Primers used to amplify mtDNA fragments of cytochrome *b* of *Paradactylodon*.

Primer Name	mtDNA fragment	Source	3'=>5' sequence
PgorgCytbF1	cytochrome *b*	This paper	CCAACTCCATCAAACATCTCTT
PgorgCytbF2_short	fragment of cyt *b*	This paper	CAGCCTTTTCATCCGTAGCC
PgorgCytb_R4	fragment of cyt *b*	This paper	GAGGGCGTCTTTAAAGGAAA
PgorgCytb_F4	fragment of cyt *b*	This paper	CCTCTAACACAGATAAAGTACCATTCC
PgorgCytb_R1	fragment of cyt *b*	This paper	CCGTCCGATATGGAGGTAGA
PgorgCytb_R2	fragment of cyt *b*	This paper	CCGCCAATTCAGGTTAAGAT
