# Supplementary material for: Shedding Light on a Secretive Tertiary Urodelean Relict: Hynobiid Salamanders (Paradactylodon persicus s.l.) from Iran, Illuminated by Phylogeographic, Developmental, and Transcriptomic Data
Source: Genes (Basel). 2019 Apr 18;10(4):306. doi: 10.3390/genes10040306 (PMC6523714; doi:10.3390/genes10040306)
Supplement: Supplementary file 1 [file genes-10-00306-s001.zip › SupplementaryMaterials_StoeckEtAl/TableS1_StoeckEtAl.pdf]

Table S1. List of samples of Iranian *Paradactylodon*; sample IDs according to Figure 1.

Locality ID	Sample ID	Locality	lat	lon	Reference
1	ZSM 821/2006_1	Iran, Gilan Province, Talysh-mountains, 12 km S Assalem, 700 m a.s.l. (series of 11 larvae), leg. J.J. & J.F. Schmidtler, 1 June 1970	37.734 N	48.916 E	Schmidtler & Schmidtler, 1971
1	ZSM 821/2006_2	Iran, Gilan Province, Talysh-mountains, 12 km S Assalem, 700 m a.s.l. (series of 11 larvae), leg. J.J. & J.F. Schmidtler, 1 June 1970	37.734 N	48.916 E	Schmidtler & Schmidtler, 1971
1	ZSM 821/2006_3	Iran, Gilan Province, Talysh-mountains, 12 km S Assalem, 700 m a.s.l. (series of 11 larvae), leg. J.J. & J.F. Schmidtler, 1 June 1970	37.734 N	48.916 E	Schmidtler & Schmidtler, 1971
1	ZSM 821/2006_4	Iran, Gilan Province, Talysh-mountains, 12 km S Assalem, 700 m a.s.l. (series of 11 larvae), leg. J.J. & J.F. Schmidtler, 1 June 1970	37.734 N	48.916 E	Schmidtler & Schmidtler, 1971
2	ZMGU1083	Iran, Mazandaran Prov., SE of Chalous city, Lashkenar village, valley of Zereshkdarreh; leg. Kaveh Samimi, larva coll. Sept. 10, 2004 in small ponds near spring	36.598 N	51.444 E	This paper
2	ZMGU1084	Iran, Mazandaran Prov., SE of Chalous city, Lashkenar village, valley of Zereshkdarreh; leg. Kaveh Samimi, larva coll. Sept. 10, 2004 in small ponds near spring	36.598 N	51.444 E	This paper
3	MMTT2123	Iran, Yeilagh-e-Sarasi, ca. 45 km SE Khalkhal, Delmadeh (Daylamdeh) village, 37°36′N, 48°31′E, 1500 m a.s.l.	37.377 N	48.788 E	Kami, 1999
3	MMTT2124	Iran, Yeilagh-e-Sarasi, ca. 45 km SE Khalkhal, Delmadeh (Daylamdeh) village, 1500 m a.s.l., leg.	37.377 N	48.788 E	Kami, 1999
3	MMTT2127	Iran, Yeilagh-e-Sarasi, ca. 45 km SE Khalkhal, Delmadeh (Daylamdeh) village, 1500 m a.s.l., leg.	37.377 N	48.788 E	Kami, 1999
3	MMTT2128	Iran, Yeilagh-e-Sarasi, ca. 45 km SE Khalkhal, Delmadeh (Daylamdeh) village, 1500 m a.s.l., leg.	37.377 N	48.788 E	Kami, 1999
3	EbhI	Iran, Khalkhal City, Ardabil Province, Karmen, near Deylamdeh village	37.377 N	48.788 E	This paper
4	Weyser	Iran, Mazandaran Province, near Veysar village, Stöck & Voitel leg., 2015	36.508 N	51.444 E	This paper
5	EbhIII	Iran, Mazandaran Province, Veysar village, Noshahr City, Zaresk-Dareh	36.482 N	51.541 E	This paper
5	EbhII	Iran, Mazandaran Province, Veysar village, Noshahr City, Zaresk-Dareh	36.482 N	51.541 E	This paper
6	MTD41098	Iran, Shirabad Cave, 5 km SE (air) of Shirabad, 60 km E (by air) of Gorgan	36.950 N	55.040 E	Stöck, 1999
6	BgA3	Iran, Shirabad Cave, 5 km SE (by air) of Shirabad, 60 km E (by air) of Gorgan	36.950 N	55.040 E	Stöck, 1999
6	BgA2	Iran, Shirabad Cave, 5 km SE (by air) of Shirabad, 60 km E (by air) of Gorgan	36.950 N	55.040 E	Stöck, 1999
6	BgA1	Iran, Shirabad Cave, 5 km SE (by air) of Shirabad, 60 km E (by air) of Gorgan	36.950 N	55.040 E	Stöck, 1999
6	DQ333822.1	Iran, Shirabad Cave, 5 km SE (by air) of Shirabad, 60 km E (by air) of Gorgan	36.950 N	55.040 E	Zhang et al. 2006
