# Supplementary figures and images for: Shedding Light on a Secretive Tertiary Urodelean Relict: Hynobiid Salamanders (Paradactylodon persicus s.l.) from Iran, Illuminated by Phylogeographic, Developmental, and Transcriptomic Data
Source: Genes (Basel). 2019 Apr 18;10(4):306. doi: 10.3390/genes10040306 (PMC6523714; doi:10.3390/genes10040306)

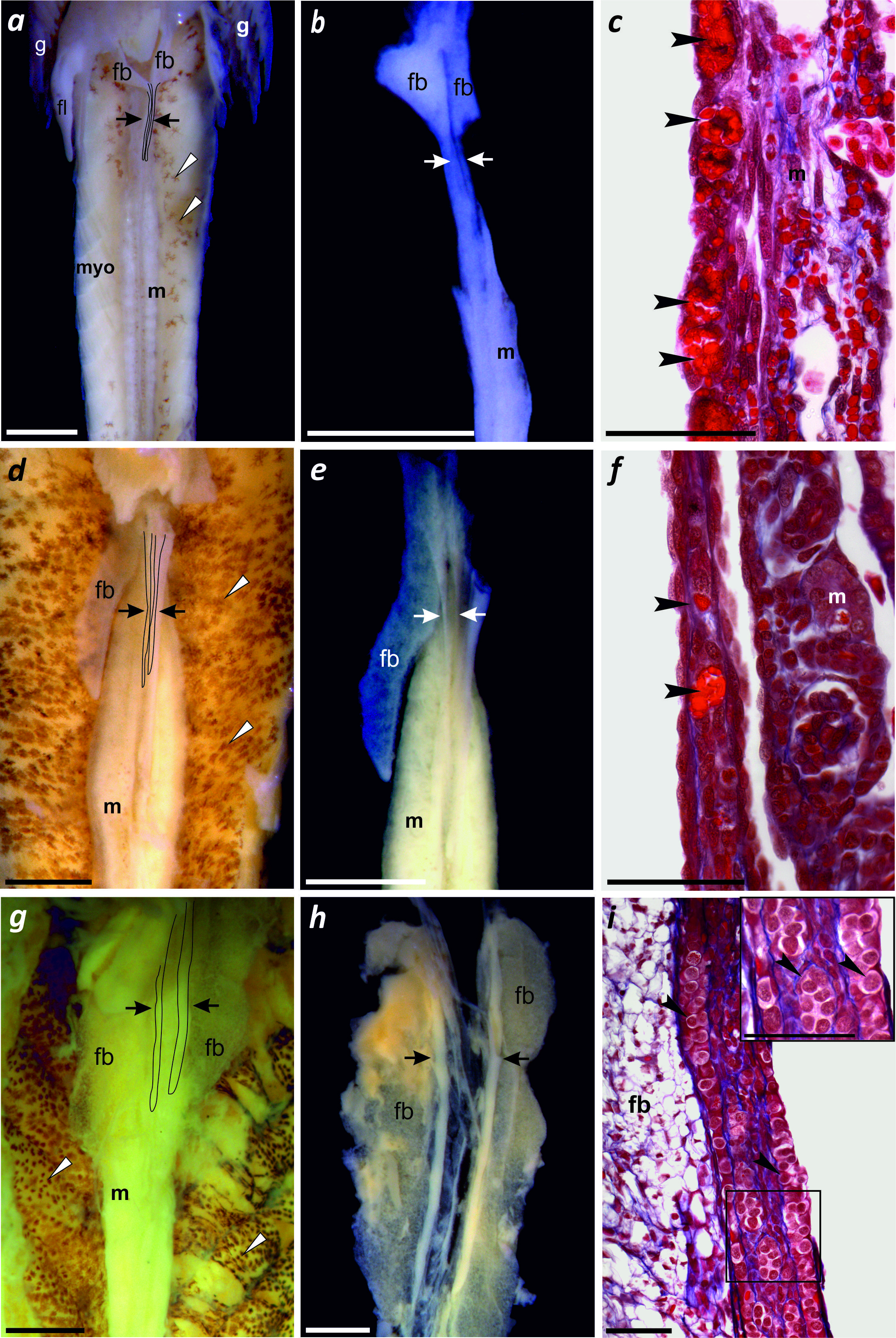

Supplement: Supplementary file 1 [file genes-10-00306-s001.zip › SupplementaryMaterials_StoeckEtAl/FigS3_StoeckEtAl.jpg]

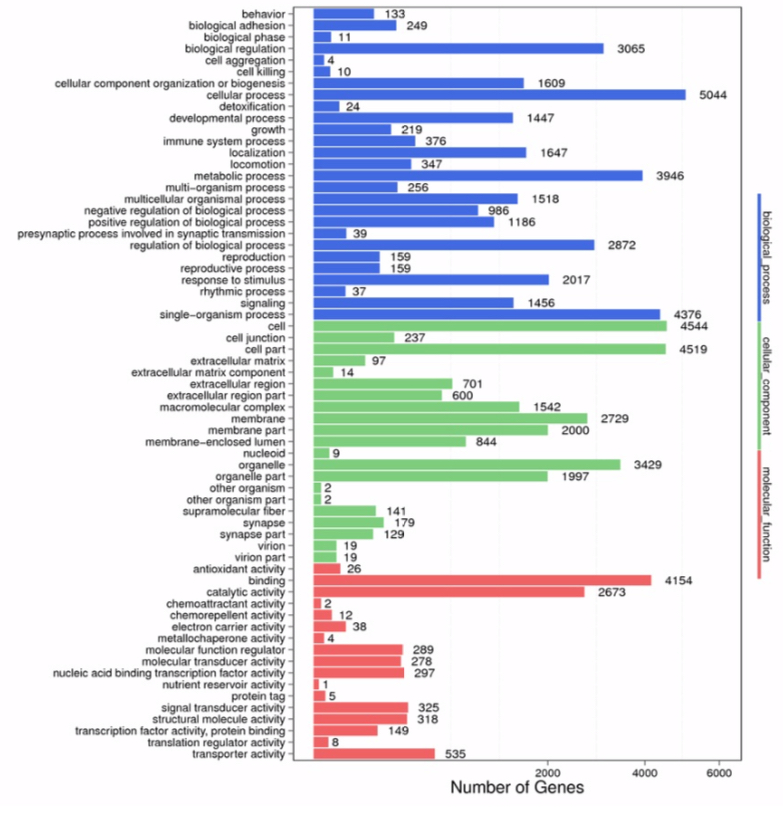

Supplement: Supplementary file 1 [file genes-10-00306-s001.zip › SupplementaryMaterials_StoeckEtAl/FigS2_StoeckEtAl.jpg]

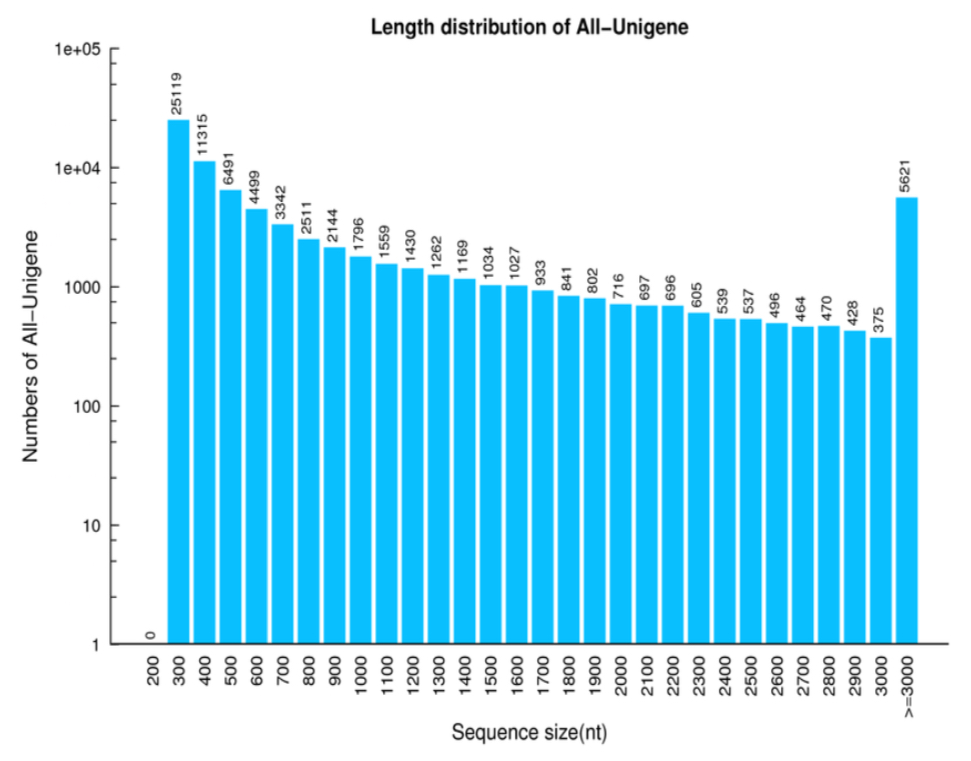

Supplement: Supplementary file 1 [file genes-10-00306-s001.zip › SupplementaryMaterials_StoeckEtAl/FigS1_StoeckEtAl.jpg]
